# Coronary Sinus Narrowing Improves Right Ventricular Function

**DOI:** 10.1016/j.jacasi.2022.03.003

**Published:** 2022-06-07

**Authors:** Tomer D. Mann, Natalia Kofman, Asaf Katz, Maayan Konigstein, Michal Laufer Perl, Meital Elbaz Zuzut, Miri Revivo, Yan Topilsky, Shmuel Banai, Ofer Havakuk

**Figure:**
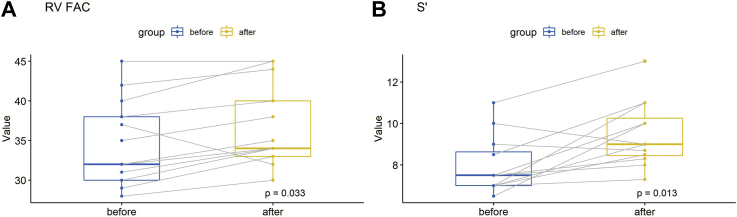

Right ventricular (RV) function affects outcome in coronary artery disease (CAD) patients.^1^ Elevating coronary sinus (CS) pressure with the use of coronary Reducer device (Neovasc) was shown to improve subendocardial blood flow in the ischemic heart, alleviate ischemia and angina, and improve left ventricular (LV) function.^2^^,^^3^ We tested whether in patients with refractory angina and ischemia, who are treated with Reducer implantation, the increased CS pressure will also improve RV performance.

We conducted a single-center, single-arm, open-labeled prospective study, enrolling consecutive patients with obstructive CAD and refractory angina despite optimal medical therapy, who were not candidates for revascularization procedures. All participants had objective evidence of reversible myocardial ischemia in technetium sestamibi scan, LV ejection fraction (LVEF) ≥35%, and no significant valvular disease. Primary pulmonary hypertension was excluded when appropriate.

All underwent full echocardiographic evaluation at baseline and 4 to 6 months following Reducer implantation. Impaired RV function was defined as the presence of ≥2 of the following: RV fractional area change (RV FAC) <35%, tricuspid annulus plane systolic excursion (TAPSE) <16 mm, peak systolic lateral tricuspid annular velocity (S') <10 cm/s and myocardial performance index (MPI) >0.44. All measurements were done in accordance with the American Society of Echocardiography Guidelines for the Echocardiographic Assessment of the Right Heart in Adults.^4^ LVEF was evaluated using the Simpson method, and LV diastolic function was evaluated by integration of mitral inflow, left atrial volume index, tissue Doppler imaging of the mitral annulus, and peak tricuspid regurgitation velocity. Images were obtained in a steady state condition by expert technicians and cardiologists blinded to the study details using the same equipment (iE33, Philips Medical Systems). No changes in medical treatment were allowed for the first 6 months post-implantation. The study was approved by the Tel Aviv Medical Center institutional ethics committee, and all participants signed an informed consent (Use of the Neovasc Coronary Sinus Reducer System for the Treatment of Refractory Angina Pectoris in Patients With Angina Class 3-4 Who Are Not Candidates for Revascularization [Reducer]; NCT01566175).

Enrolled were 25 patients. Mean age was 67 ± 9 years, 84% men, mean CCS (Canadian Cardiovascular Society) 3.4 ± 0.8. Baseline RV dysfunction was present in 14 patients (56%). Patients with and without RV dysfunction had similar baseline characteristics in terms of age (66 ± 10.8 vs 68 ± 7.1 years; *P* = 0.63), sex (85% vs 88% men; *P* = 1.00), LVEF (48 ± 9.1% vs 49 ± 5.3%; *P* = 0.64), right coronary artery (RCA) involvement (4 vs. 4; *P* = 1.00), regional LV ischemic distribution, diastolic function, and cardiovascular risk profile. Following Reducer implantation, a similar degree of angina relief was observed in patients with and without RV dysfunction.

**Figure 1:**
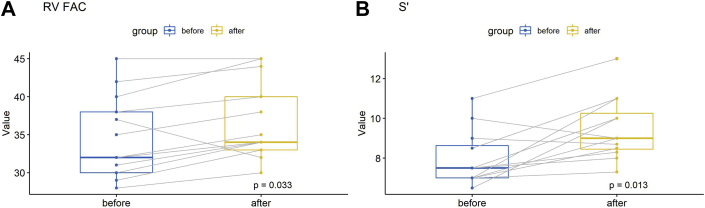
Coronary Reducer Implantation Effect on RV Function Of 25 severe coronary artery disease patients, 14 had baseline RV dysfunction. Reducer implantation (known to ameliorate myocardial ischemia) was associated with an improvement in several RV function indices (eg, RV FAC improved from 34.1 ± 5.4% to 36.7 ± 5.1%; *P* = 0.033 and S' improved from 8 ± 1.2 cm/s to 9.5 ± 1.5 cm/s; *P* = 0.01), a finding that might contribute to patients' clinical stabilization. FAC = fractional area change; RV = right ventricular.

Improvement in RV function indices after Reducer implantation was shown in the entire cohort but reached statistical significance in the subgroup of patients with baseline RV dysfunction (S' 8 ± 1.2 cm/s to 9.5 ± 1.5 cm/s; *P* = 0.01; RV FAC 34.1 ± 5.4% to 36.7 ± 5.1%; *P* = 0.033; MPI 0.56 ± 0.07 to 0.49 ± 0.05; *P* = 0.036, and TAPSE from 15.8 ± 3.2 cm to 16.2 ± 2.9 cm; *P* = 0.001, before and after Reducer implantation, respectively) (Figure 1). The improvement in RV function was not associated with either LV systolic or diastolic function change (*P* for interaction = 0.6). However, lateral LV wall ischemia was associated with an improvement in several RV function indices (*P* for interaction = RV FAC 0.024, S' 0.011 cm/s, and MPI 0.008, respectively).

Though physiological differences in afterload, wall stress, and myocardial perfusion allow the RV to better recover from ischemic injury,^5^^,^^6^ its function is jeopardized in the presence of ischemia. Our study demonstrates that in severe CAD patients, CS narrowing with the use of coronary Reducer may induce RV function recovery. These findings correlate with previous reports showing an improvement in LV function in similar patients.^3^ However, the improvement shown in RV function in our study was not associated with LV systolic or diastolic function changes and implies that the RV may directly benefit from the improved myocardial perfusion induced by the Reducer device. Furthermore, RCA involvement in our cohort was not associated with the effect of Reducer on RV function, corresponding with a recent publication showing that the Reducer is effective also when a total occlusion of the RCA was found.^7^ Small-vessel disease is prevalent in CAD patients, and though RV ischemia was not directly evaluated in our study, it might be presumed that increased CS pressure with the Reducer device induces dilatation of small resistant arterioles, causing redistribution of blood to ischemic RV subendocardium. This, in turn, may lead to improved myocardial function.

Our study is limited by its small size, the use of 2-dimensional echocardiography as a single method for RV function evaluation and the lack of direct assessment of RV ischemia. Therefore, our results should be seen as hypothesis-generating and warrant repeated investigation in a larger cohort and using other methods for evaluating RV ischemia and function. Nevertheless, our findings suggest that in this small group of severe CAD patients, coronary Reducer implantation was associated with RV function improvement, a result that might contribute to patients’ clinical stabilization.
